# Seroprevalence of antibodies to dengue and chikungunya viruses in Thailand

**DOI:** 10.1371/journal.pone.0180560

**Published:** 2017-06-29

**Authors:** Sompong Vongpunsawad, Duangnapa Intharasongkroh, Thanunrat Thongmee, Yong Poovorawan

**Affiliations:** Center of Excellence in Clinical Virology, Faculty of Medicine, Chulalongkorn University, Bangkok, Thailand; Instituut voor Tropische Geneeskunde, BELGIUM

## Abstract

The abundance of *Aedes* mosquito species enabled widespread transmission of mosquito-borne chikungunya virus (CHIKV) and dengue virus (DENV) in Southeast Asia. Periodic seroprevalence surveys are therefore necessary to assess the viral burden in the population and the effectiveness of public health interventions. Since the current seroprevalence for CHIKV and DENV in Thailand are unknown, we evaluated evidence of past infection among Thais. Eight-hundred and thirty-five serum samples obtained from individuals living in central and southern Thailand were assessed for anti-CHIKV and anti-DENV IgG antibodies using commercial enzyme-linked immunosorbent assays. Overall, 26.8% (224/835) of individuals were seropositive for CHIKV, the majority of whom were also DENV-seropositive (91.1%, 204/224). Approximately half of all adults in their fifth decade of life had attained CHIKV seropositivity. Children under 15 years of age in southern Thailand were significantly more likely to be CHIKV-seropositive compared to those residing in central Thailand. In contrast, 79.2% (661/835) of Thais were DENV-seropositive, 30.9% (204/661) of whom also had antibodies to CHIKV. CHIKV/DENV dual seropositivity among Thais was 24.4% (204/835). The age-standardized seroprevalence for DENV was three times that of CHIKV (80.5% vs. 27.2%). Relatively high CHIKV seroprevalence among adults living in central Thailand revealed an under-recognized CHIKV burden in the region, while the low-to-moderate transmission intensity of DENV (seroprevalence <50% at 9 years) is expected to reduce the impact of DENV vaccination in Thailand. This most recent seroprevalence data provide serological baselines for two of the most common mosquito-borne viruses in this region.

## Introduction

Dengue burden on the world’s population is estimated at approximately 60 million symptomatic infections resulting in 10,000 deaths each year [1]. Although mosquito-borne viruses have long been endemic in Southeast Asia, the dengue virus (DENV) outbreak described in 1958 concomitant with the first identification of chikungunya virus (CHIKV) in Thailand increased the awareness of arboviruses responsible for acute febrile illness in the region [2,3]. Infection caused by DENV and CHIKV presents similar clinical symptoms and are sometimes difficult to differentiate [4]. Typical symptoms of DENV infection are fever, rash, headache, myalgia, while CHIKV infection produces an additionally more pronounced musculoskeletal and neuropathic pain [5,6]. Complications from DENV infection can lead to life-threatening dengue hemorrhagic fever and dengue shock syndrome, both of which necessitate careful management of care. Meanwhile, CHIKV infection may result in debilitating arthralgia and acute and chronic arthritis long after an individual has recovered from primary infection. Consequently, accurate diagnosis can be difficult without molecular diagnostics especially in many resource-limited settings where arboviruses are endemic [7].

The tropical climate of Thailand is conducive to the spread of *Aedes aegypti* and *Aedes albopictus*, the principal vectors in the transmission of CHIKV and DENV. CHIKV exists in several lineages, which are East/Central/South African (ECSA), West African, Asian, and the proposed Indian Ocean lineage [8]. Four serotypes of DENV found in Thailand vary in predominance in a given year [9]. While outbreaks caused by CHIKV have been sporadic and somewhat regionally restricted, widespread DENV infection occurs throughout the year with cyclical intervals of epidemic years defined by very high incidence (>100,000 cases) during the rainy months [10–13].

The lack of current seroepidemiological data in Thailand presents a significant knowledge gap in DENV and CHIKV disease burden and susceptibility in a region of significant commerce and migration convergence within Southeast Asia. While pre-existing immunity towards CHIKV does not appear to worsen reinfection by another CHIKV lineage, heterotypic DENV reinfection can increase the risk of severe clinical outcome resulting from antibody-dependent enhancement of infection [6,14]. Hence, periodic seroprevalence surveys are important in order to evaluate immunity in a population and appropriate public health interventions such as vaccination. Here, we assessed past exposure to DENV and CHIKV by evaluating pre-existing IgG antibodies to these viruses in Thai children and adults.

## Materials and methods

### Study population

**Fig 1:**
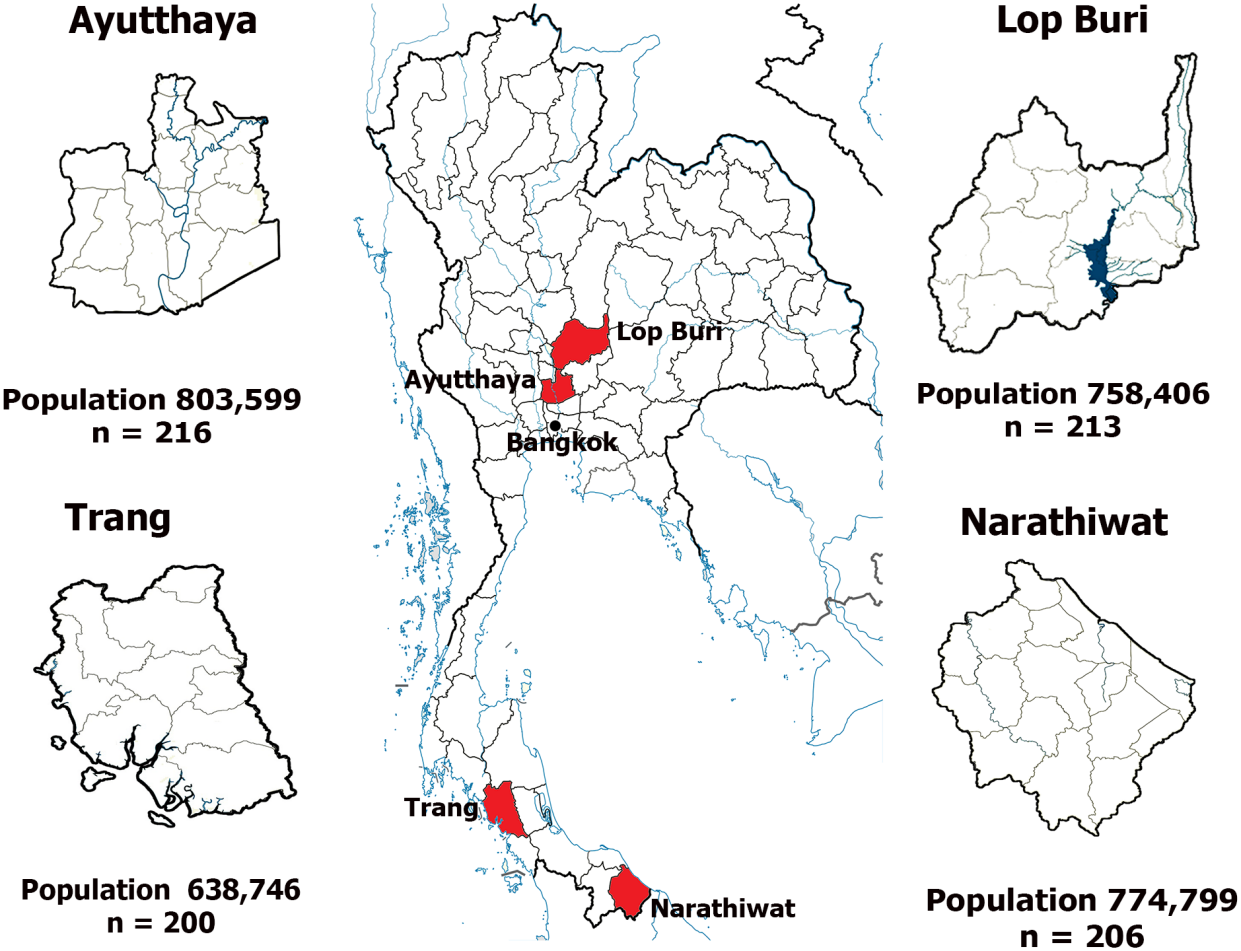
Map of Thailand shows four provinces from which seroprevalence data were derived. Bangkok is indicated as reference. Population was based on the 2014 census data from the Official Statistics Registration System (http://stat.dopa.go.th/stat/statnew/upstat_age.php).

This study utilized residual sera from a previously published seroprevalence survey of viral hepatitis burden in Thailand [15]. Serum samples were from individuals 6 months to 60 years who sought scheduled health check-ups or outpatient clinic care at 4 provincial hospitals located in Ayutthaya (n = 216), Lop Buri (n = 213), Narathiwat (n = 206), and Trang (n = 200) between April and October 2014. A total of 835 randomly chosen residual blood samples were tested. Provinces were selected for their similar population size and healthcare infrastructure capacity. Ayutthaya and Lop Buri are adjacent provinces in central Thailand 50 to 90 kilometers north of the capital city Bangkok, respectively (Fig 1). Agriculture, small factories, and animal farming predominate. Trang and Narathiwat are approximately 800 and 1,100 kilometers south of Bangkok, respectively. These provinces are relatively forested and interspersed with Para rubber plantations. The Institutional Review Board of the Faculty of Medicine, Chulalongkorn University approved this study (IRB 435/57) and waived the need for consent because the samples were de-identified and anonymous. Permission to use these stored anonymous residual sera were granted by the director of King Chulalongkorn Memorial Hospital.

### Serological analysis

Anti-IgG antibodies against CHIKV and DENV were assessed using commercial enzyme-linked immunosorbent assays on 96-well microplates (catalog numbers EI293a-9601G and EI266b-9601G, EUROIMMUN, Lübeck, Germany) according to the manufacturer’s instructions. Briefly, serum samples diluted 1:100 were added to the microplates containing either recombinant structural protein of CHIKV or purified DENV particles, and colorimetric output was evaluated in a microplate reader. OD ratios were extrapolated and antibody values ≥20 relative units/ml were considered seropositive.

### Data analysis

The DENV and CHIKV IgG prevalence among residents were weighed to adjust for age and gender distribution in the population by direct method using the sampling weights from the 2014 census data. Association of categorical variables with positive serology was evaluated by binary logistic regression using univariate analysis. Data were statistically analyzed with SPSS software version 22. *P* value <0.05 was considered statistically significant.

Seroprevalence level among children 9 years of age (SP9) was extrapolated from the seroprevalence data by plotting the age-stratified DENV seroprevalence rates and subjecting the data to a best-fit polynomial curve using MATLAB (MathWorks, Natick, MA). DENV transmission intensity settings were defined as very low (SP9 = 10%), low (SP9 = 30%), moderate (SP9 = 50%), high (SP9 = 70%), and very high (SP9 = 90%) according to the World Health Organization [16–17].

## Results

### Demographic data

In this study, the cohort comprised 47.8% men and 52.2% women. Their age ranged from 6 months to 60 years with a mean of 29.7 years and a median of 31 years. There was no statistically significant differences in the gender (*p* = 0.097) or age distribution of the individuals (*p* = 0.457) between central and southern regions.

### CHIKV-specific IgG prevalence

**Fig 2:**
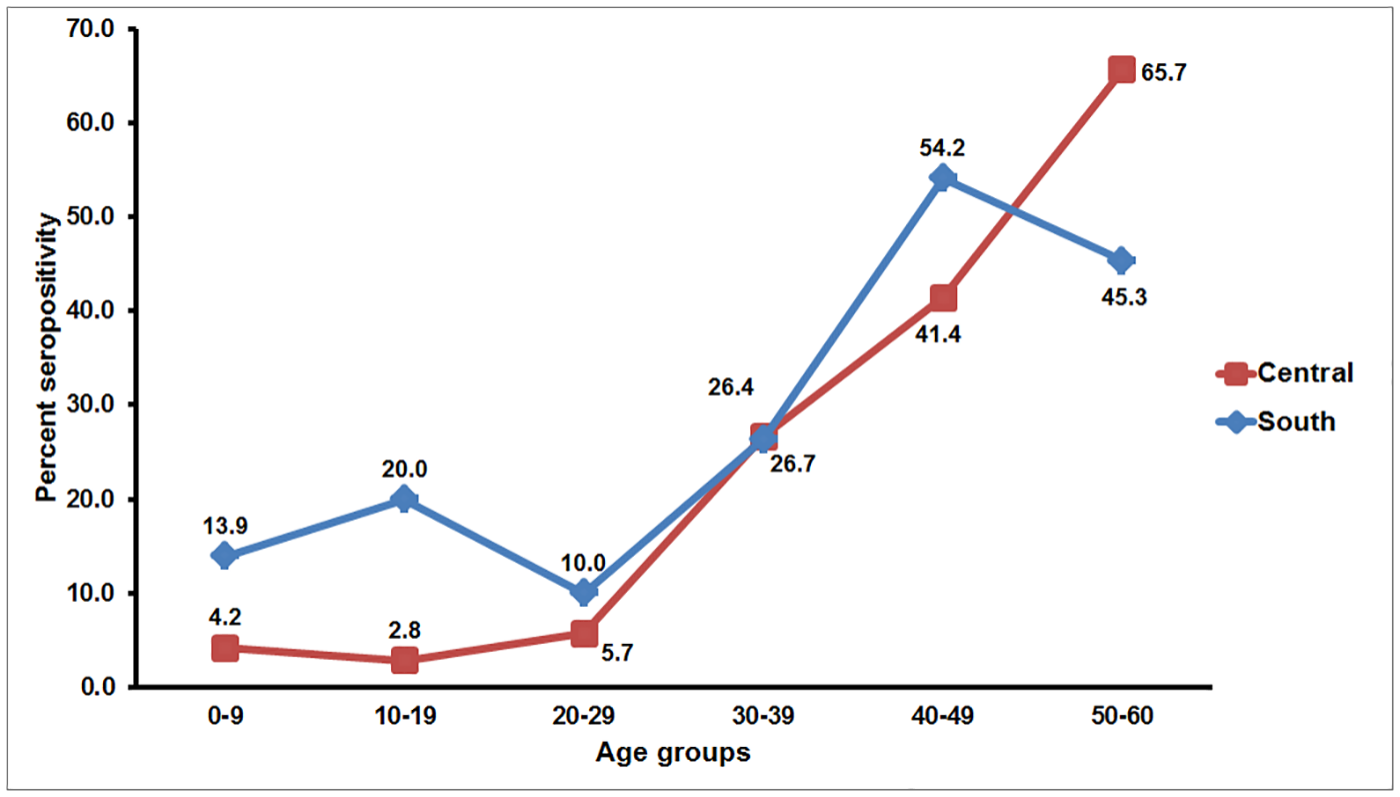
Age group-specific CHIKV seroprevalence in Thailand by region. The line graphs represent regional CHIKV seroprevalence (y-axis) relative to age groups (x-axis). Seropositivity rates for each age group are indicated on the graphs.

Overall, individuals who tested positive for anti-CHIKV IgG antibody comprised 26.8% (224/835). The age-standardized seroprevalence was similar at 27.2%. There were no statistically significant differences in the seropositive rates based on gender (26.1% male versus 27.5% female) or region (24.2% central versus 29.6% south) (Table 1). However, seroprevalence increased with increasing age and approximately half of adults in their 50’s had seroconverted (Fig 2). Since seroprevalence appeared to rise dramatically around age 30, we stratified the cohort into four age groups to facilitate data analysis. Individuals 31 years of age or older were more likely to possess antibodies than those <15 years old (*p* < 0.001). Although regional differences in the seroprevalence rates in men were not statistically significant, individuals younger than 15 years of age from the south were significantly more likely to be seropositive than those from central Thailand (Table 2).

**Table 1:** Seroprevalence of anti-CHIKV IgG antibody.

Characteristics	Total	Seropositive (%)	Crude OR (95% CI)	*p* value
**Gender**				
**Male**	399	104 (26.1)	-	-
**Female**	436	120 (27.5)	1.1 (0.8–1.5)	0.635
**Age**				
**<15**	232	24 (10.3)	-	-
**15–30**	180	17 (9.4)	0.9 (0.5–1.7)	0.762
**31–45**	217	69 (31.8)	4 (2.4–6.7)	<0.001*
**>45**	206	114 (55.3)	10.7 (6.5–17.8)	<0.001*
**Region**				
**Central**	429	104 (24.2)	-	-
**South**	406	120 (29.6)	1.3 (1.0–1.8)	0.084

* Denote statistical significance.

**Table 2:** Analysis of anti-CHIKV IgG antibody by region.

Characteristics	South		Central		OR (95% CI)	*p* value
	Total	Seropositive (%)	Total	Seropositive (%)		
**Gender**						
**Male**	182	56 (30.8)	217	48 (22.1)	1.6 (1.0–2.5)	0.051
**Female**	224	64 (24.6)	212	56 (26.4)	1.1 (0.7–1.7)	0.614
**Age**						
**<15**	118	21 (17.8)	114	3 (2.6)	8 (2.3–27.7)	0.001*
**15–30**	74	9 (12.2)	106	8 (7.5)	1.7 (0.6–4.6)	0.302
**31–45**	113	39 (34.5)	104	30 (28.8)	1.3 (0.7–2.3)	0.371
**>45**	101	51 (50.5)	105	63 (60.0)	0.7 (0.4–1.2)	0.171

* Denote statistical significance.

### DENV-specific IgG prevalence

**Fig 3:**
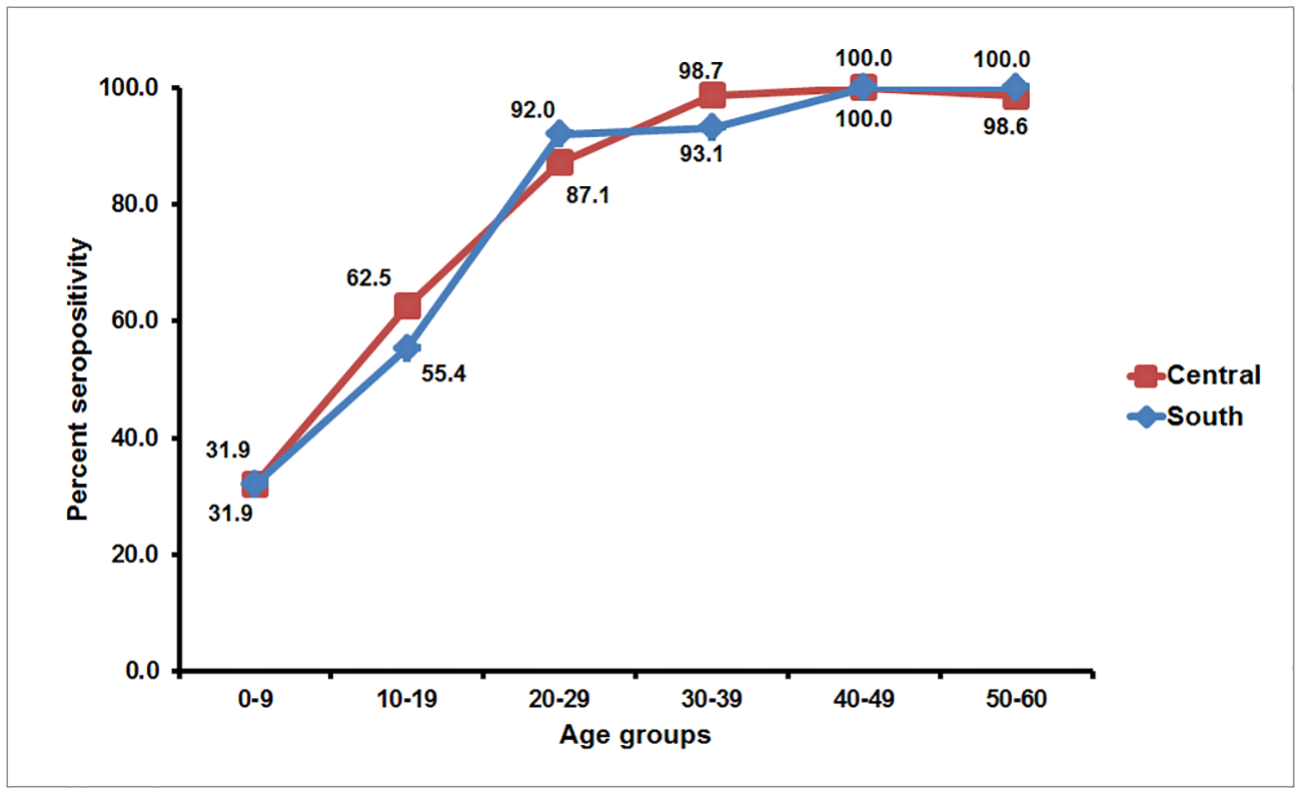
Age group-specific anti-DENV IgG seroprevalence in Thailand by region. The line graphs represent regional DENV seroprevalence (y-axis) relative to age groups (x-axis). Seropositivity rates for each age group are indicated on the graphs.

The overall seropositive rate for anti-DENV IgG antibody was 79.2% (661/835), which was similar to the age-standardized seroprevalence of 80.5%. However, this was three times higher than that of CHIKV. Seroprevalence did not significantly differ based on gender and region of residence, but increasing age appeared to be associated with increased seropositivity (*p* < 0.001) (Table 3). To better compare our finding with published data from the dengue vaccine trial in Southeast Asia in which Thailand was one of the study sites [18], individuals were stratified into age groups <9, 9 to 45, and >45 years of age. Overall, approximately 30% of children younger than 9 years of age possessed anti-DENV IgG antibody, while seroconversion rate exceeded >99% in individuals older than 45 years of age (Fig 3). Unlike CHIKV, there were no significant differences in the regional seroprevalence among age groups (Table 4).

**Table 3:** Seroprevalence of anti-DENV IgG antibody.

Characteristics	Total	Seropositive (%)	Adjusted OR (95% CI)	*p* value
**Gender**				
**Male**	399	306 (76.7)	-	-
**Female**	436	355 (81.4)	1.3 (1.0–1.9)	0.093
**Age**				
**<9**	133	40 (30.1)	-	-
**9–45**	496	416 (83.9)	12.1 (7.8–18.8)	<0.001*
**>45**	206	205 (99.5)	476.6 (64.5–3519.6)	<0.001*
**Region**				
**Central**	429	342 (79.7)	-	-
**South**	406	319 (78.6)	0.9 (0.7–1.3)	0.683

* Denote statistical significance.

**Table 4:** Analysis of anti-DENV IgG antibody by region.

Characteristics	South		Central		OR (95% CI)	*p* value
	Total	Seropositive (%)	Total	Seropositive (%)		
**Gender**						
**Male**	182	134 (73.6)	217	172 (79.3)	0.7 (0.5–1.2)	0.186
**Female**	224	185 (82.6)	212	170 (80.2)	1.2 (0.7–1.9)	0.52
**Age**						
**<9**	67	20 (29.9)	66	20 (30.3)	1.0 (0.5–2.1)	0.955
**9–45**	238	198 (83.2)	258	218 (84.5)	0.9 (0.6–1.5)	0.694
**>45**	101	101 (100)	105	104 (99.0)	-	0.997

The licensed dengue vaccine is indicated for children 9 years of age or older living in DENV endemic area, but it is not recommended when the age group seroprevalence among 9 year-olds (SP9) is below 50% [16]. We therefore determined the SP9 in our cohort to assess the feasibility of a routine DENV vaccine administration in Thai children (S1 Fig). Extrapolation of age-stratified seroprevalence showed SP9 = 49%, which reflects a low-to-moderate DENV transmission intensity of the population in this study.

### Evidence of dual anti-CHIKV and anti-DENV antibodies

To determine the overlapping endemicity between CHIKV and DENV, we examined the presence of IgG antibodies to both viruses in our cohort. We found that 24.4% (204/835) of all individuals in this study possessed both anti-CHIKV and anti-DENV IgG antibodies. Among 224 CHIKV-seropositive individuals, 91.1% (204/224) were also DENV-seropositive. In contrast, 30.9% (204/661) of individuals with anti-DENV IgG antibodies had anti-CHIKV IgG antibodies. Most dual seropositive individuals tended to be adults, but there were no statistically significant differences based on gender (*p* = 0.121) or region of residence (*p* = 0.052).

## Discussion

Awareness of the scope of CHIKV and DENV endemicity in Southeast Asia is important in assisting the diagnosis and control of these mosquito-borne viruses. Although they account for significant morbidity in the region, the extent of the viral burden is often obscured by inadequate surveillance and clinical misdiagnosis. Past seroprevalence studies over several decades have demonstrated that the presence of anti-CHIKV and anti-DENV antibodies increased with age [19–20]. For CHIKV, which is the only circulating alphavirus in Thailand, seroprevalence reached approximately 50% by 45 years of age in Thais residing in central Thailand [19]. Almost 40 years later, our study showed that the current CHIKV seropositive rate in this region has noticeably decreased. Meanwhile, the observation that individuals younger than 15 years of age in southern Thailand were more likely to be CHIKV seropositive compared to their counterparts in central Thailand may in part be due to the sharp increase in the antibody conversion rate attributed to the well-documented CHIKV outbreaks in southern Thailand in 1995 [21] and again in 2008 [22–23]. Surprisingly, higher seroprevalence in central rather than southern Thailand among 50–59 year-olds suggests that CHIKV circulation may have been under-recognized in the past, despite its reported absence in central Thailand between 1979 and 1982 [24]. Although outbreaks of CHIKV of Asian lineage occurred prior to 1990, ECSA lineage appears to predominate in recent years [12–13]. Limited antigenic diversity of CHIKV likely affords heterologous protection to infection by different CHIKV lineages in previously infected population [6].

While the age-standardized seroprevalence for DENV was three times that of CHIKV and the majority of individuals attained anti-DENV IgG antibodies by the age of 30 years, less than half of young children demonstrated DENV seropositivity in our study. This is different from what was observed decades ago whereby DENV seroconversion occurred early in life and most Thais possessed antibodies against at least one dengue serotype by the age of 20 years [19]. The decreasing DENV transmission intensity in Thailand is exemplified by the recent finding in Rayong province 100 kilometers southeast of Bangkok, which reported a decrease in SP9 from approximately 70% in 1980 down to 45% in 2010 [20]. The increasing age at which individuals are DENV-seropositive parallels the present-day shifting of clinically apparent dengue disease from children to young adults in the general population as improved socioeconomic development reduced the risk of DENV exposure [11]. In addition, lower birth rate has shifted the median age of the population and subsequently delayed the age of secondary infection, which is associated with dengue disease more often now in adults than in children [25].

Co-infection and co-distribution of DENV and CHIKV have been reported in Thailand [7,26–28], so it was not surprising that about one-quarter of our cohort demonstrated dual seropositivity, most of whom were older adults. However, this was significantly higher than the serosurvey results conducted in the early 1960’s, which revealed that 13% of Thais had antibodies to both DENV and CHIKV [29]. Although there is no conclusive evidence to suggest that concurrent co-infection of CHIKV and DENV results in more severe symptoms than monotypic infection alone, clinical complications may depend largely on whether DENV infection was secondary [7].

Admittedly, comparisons of seroprevalence among studies in different countries are complicated by the differences in the demographics of the population studied, stratification of age groups, viral antibody detection used, and study approach. In comparison to neighboring Malaysia, the CHIKV seroprevalence in this study is higher than that reported among healthy Malaysian adults ≥35 years old (5.9%) residing in outbreak-free areas [30]. In contrast, regional DENV seroprevalence rates have been comparable. For example, approximately 92% of Malaysian adults ≥35 years old are reportedly DENV seropositive [31]. Among Singaporeans 16–60 and 18–79 years old surveyed in 2010, DENV seroprevalence rates were 50.8% [32] and 54.4% [33], respectively. Both rates demonstrated a significant decrease in the DENV burden from the previous survey (63.1%) conducted in 2004 [34], a trend that is also seen in Thailand. However, unlike those studies in which men were more likely to have been exposed to DENV than women, gender was not associated with higher seroprevalence in our cohort.

It should be noted that as with most commercial antibody-based assays, cross-reactivity of anti-DENV antibody with other flaviviruses cannot be excluded. Additionally, since archived serum samples were used, adults older than 60 years were not available for inclusion. It would have been interesting to determine viral RNA or virus-specific IgM in the serum samples, which could have revealed potential active viremia or acute infection. Measurement of antibody prevalence did not distinguish for specific DENV serotype nor different CHIKV lineages, and using serology alone may sometimes underestimate the true incidence of immunity, especially in individuals with low antibody titers. Despite these limitations, this study provides the most recent seroprevalence data for two of the most common mosquito-borne viruses in Southeast Asia.

Accurate seroprevalence data have important implications for public health policy and effectiveness of any vaccination initiatives. If widespread herd immunity for CHIKV exists, previously infected individuals will be protected from reinfection, which would dampen future CHIKV outbreak. Seroprevalence surveys conducted shortly after CHIKV outbreaks in several countries suggest that widespread infection generally declined after ~70% of the individuals have developed antibodies to CHIKV [35]. For DENV, however, cross-protection from heterologous infection is limited. In fact, antibody-dependent enhancement of DENV infection can result in severe dengue complications and may enhance disease upon infection by another flavivirus such as Zika virus [36–37]. Of particular concern is the DENV vaccine because current evidence suggests vaccination could increase the risk of developing severe disease in DENV immuno-naïve children [18,38]. In contrast, existing data have so far provided no support of immune-mediated severe disease in CHIKV infection should CHIKV vaccine become commercially available [39–40].

As a result of demographic shifts in developing countries with improved socio-economic development and lower birth rate, young Thai children are increasingly likely to have never experienced DENV infection [25]. Since DENV baseline seropositivity of the population being vaccinated is an important determinant of vaccine efficacy, children who are seronegative at the time of first vaccination may be primed for future risk of severe dengue illness especially in regions of low to moderate (SP9 = 30%-50%) and even moderate to high (SP9 ≥ 50%) endemicity [17]. Due to low vaccine efficacy in age group 2–5 years old and highest vaccine efficacy in age group 12–14 years old, the World Health Organization has recommended the lower limit of vaccine indication at 9 years old [16]. Even if the age in which DENV seroprevalence of 70% was a chosen prerequisite for vaccine administration, our data suggest that such vaccination program might have to wait until Thais reached their late teens or around the time when they are nearly completing secondary schools. As higher vaccine efficacy was achieved when vaccinating DENV-seropositive individuals, and seropositivity correlated with increasing age, routine dengue immunization in Thai children will require extremely careful consideration and forethought.

## Supporting information

S1 Fig Age-stratified DENV seroprevalence and estimate of SP9. (TIF)

S1 File ELISA data obtained from this study. (PDF)
