# Supplementary figures and images for: Seroprevalence of antibodies to dengue and chikungunya viruses in Thailand
Source: PLoS One. 2017 Jun 29;12(6):e0180560. doi: 10.1371/journal.pone.0180560 (PMC5491253; doi:10.1371/journal.pone.0180560)

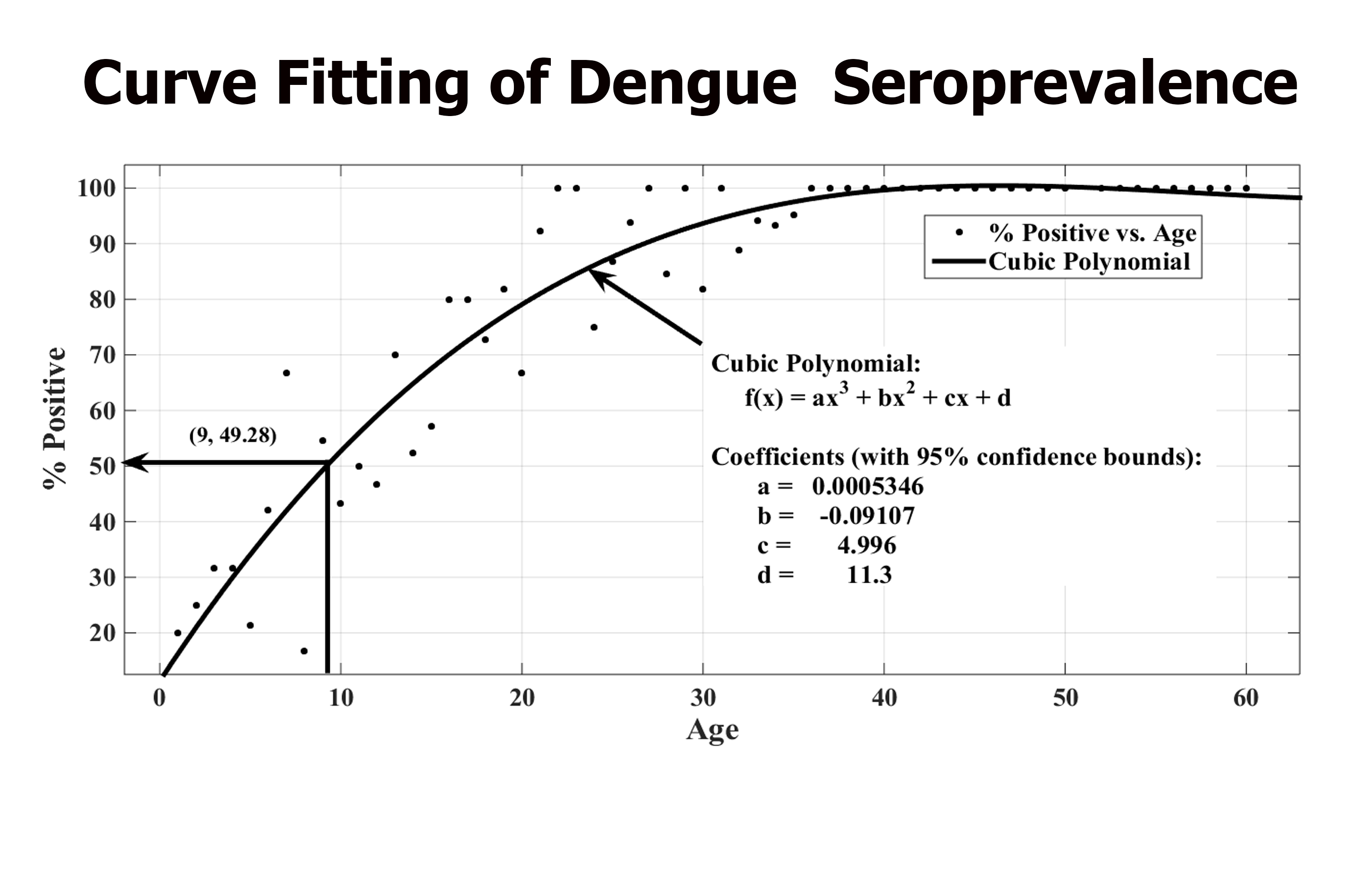

Supplement: S1 Fig — (TIF) [file pone.0180560.s001.tif]
