# Supplementary material for: Seroprevalence of antibodies to dengue and chikungunya viruses in Thailand
Source: PLoS One. 2017 Jun 29;12(6):e0180560. doi: 10.1371/journal.pone.0180560 (PMC5491253; doi:10.1371/journal.pone.0180560)
Supplement: S1 File — (PDF) [file pone.0180560.s002.pdf]

code	M/F	age	OD
ST0327	M	1	0.166
ST0194	M	2	0.228
ST0019	M	3	0.131
ST0278	F	4	0.108
ST0279	F	6	2.278
ST0280	F	8	0.268
ST0491	M	4	0.064
ST0492	F	5	0.127
ST0439	F	9	1.902
ST0563	F	9	0.179
ST705	M	3	0.114
ST688	M	1	0.154
ST679	M	1	0.077
ST666	F	6	0.069
ST682	M	5	0.136
ST709	M	0	0.063
ST703	M	1	0.112
ST702	M	7	0.078
ST667	F	0	0.107
ST681	F	2	0.606
ST668	F	2	0.085
ST665	M	3	0.061
ST712	M	4	0.099
ST675	F	9	0.064
ST683	F	3	0.138
ST707	F	2	0.106
ST671	F	3	0.101
ST695	F	3	0.089
ST692	M	0	0.095
ST684	F	1	0.161
ST677	F	4	0.105
ST696	M	3	0.085
ST669	F	2	0.066
ST680	F	2	0.127

code	M/F	age	OD
ST0298	F	10	0.219
ST0411	F	18	0.126
ST0404	F	19	0.123
ST0015	F	19	0.248
ST0024	M	10	0.133
ST0264	M	14	0.133
ST0268	M	14	0.124
ST0289	M	14	1.694
ST0025	M	12	0.177
ST0048	M	19	0.135
ST0193	M	19	1.399
ST0507	M	18	0.106
ST0508	F	18	1.571
ST0509	F	18	0.269
ST0523	F	17	0.175
ST0524	F	17	0.113
ST0525	F	19	0.098
ST0526	F	19	0.296
ST0546	F	19	0.113
ST0553	F	19	0.2
ST0578	M	15	0.191
ST678	F	10	0.093
ST689	F	11	0.095
ST690	F	13	0.06
ST706	M	11	0.126
ST708	M	13	0.059
ST713	M	12	0.092

code	M/F	age	OD
ST0272	F	20	0.192
ST0029	F	21	2.196
ST0033	F	21	0.111
ST0020	F	24	0.274
ST0208	F	24	0.174
ST0040	F	25	0.164
ST0124	F	25	0.113
ST0140	F	25	0.142
ST0141	F	25	0.136
ST0218	F	25	0.135
ST0206	F	26	1.408
ST0211	F	26	2.046
ST0424	F	26	0.203
ST0430	F	26	0.175
ST0021	F	27	0.137
ST0131	F	28	0.225
ST0207	F	28	0.126
ST0216	F	28	0.165
ST0126	F	29	0.208
ST0135	F	29	0.133
ST0221	M	26	0.083
ST0239	M	26	0.132
ST0306	M	26	0.162
ST0326	M	26	0.149
ST0362	M	26	0.124
ST0285	M	27	0.089
ST0235	M	28	0.116
ST0331	M	28	0.096
ST0367	M	28	0.117
ST0371	M	28	0.112
ST0049	M	21	0.165
ST0348	M	21	0.161
ST0253	M	24	0.137
ST0204	M	25	0.113
ST0309	M	25	0.264
ST0393	M	25	0.106
ST0277	M	26	0.143
ST0284	M	26	0.323

code	M/F	age	OD
ST0014	F	31	0.119
ST0159	F	31	0.13
ST0246	F	32	0.08
ST0247	F	32	0.363
ST0359	F	33	0.101
ST0360	F	33	0.131
ST0315	F	34	0.374
ST0397	F	34	0.134
ST0421	F	35	0.1
ST0125	F	36	0.222
ST0304	F	37	0.079
ST0313	F	37	0.167
ST0138	F	39	0.171
ST0224	F	39	0.214
ST0433	F	30	0.129
ST0422	F	33	0.329
ST0363	M	30	0.129
ST0399	M	30	1.897
ST0283	M	33	0.127
ST0034	M	34	0.086
ST0243	M	34	0.866
ST0254	M	34	0.15
ST0023	M	35	0.099
ST0400	M	36	0.229
ST0409	M	36	0.098
ST0002	M	37	0.371
ST0257	M	37	0.169
ST0353	M	37	1.407
ST0333	M	38	1.112
ST0249	M	39	0.123
ST0406	M	39	0.186
ST0506	F	33	0.097
ST0510	M	39	0.212
ST0560	M	35	0.159
ST0245	F	31	0.269
ST0290	F	37	0.066

code	M/F	age	OD
ST0310	F	40	0.208
ST0176	F	41	1.143
ST0220	F	41	0.137
ST0223	F	42	1.561
ST0428	F	42	0.157
ST0312	F	45	0.127
ST0378	F	45	0.16
ST0215	F	46	1.193
ST0320	F	46	0.394
ST0318	F	48	0.784
ST0347	F	48	0.197
ST0425	F	49	1.137
ST0432	F	49	0.08
ST0250	M	40	0.957
ST0382	M	40	0.103
ST0036	M	41	1.245
ST0256	M	41	0.491
ST0405	M	41	0.115
ST0037	M	42	0.086
ST0164	M	45	0.842
ST0394	M	45	0.273
ST0395	M	47	1.817
ST0303	M	49	0.166
ST0580	F	49	0.15
ST0500	M	42	1.073
ST0496	F	45	0.568
ST0437	F	43	0.145
ST0449	F	41	1.069
ST0490	M	40	0.273
ST0522	M	40	0.317
ST0511	M	46	0.106
ST0452	M	43	0.597
ST0434	M	49	0.858
ST0273	F	43	0.267

code	M/F	age	OD
ST0032	F	51	0.103
ST0162	F	51	0.936
ST0031	F	53	0.963
ST0058	F	53	0.064
ST0232	F	55	0.406
ST0314	F	55	0.539
ST0236	F	56	0.233
ST0060	F	58	0.16
ST0407	F	58	0.146
ST0372	F	59	0.202
ST0292	F	60	0.208
ST0412	F	60	0.562
ST0258	M	50	0.157
ST0375	M	50	0.994
ST0200	M	51	0.697
ST0139	M	54	0.098
ST0255	M	54	1.381
ST0351	M	55	0.421
ST0271	M	56	0.083
ST0061	M	57	0.161
ST0350	M	57	0.471
ST0055	M	59	0.103
ST0064	M	59	0.289
ST0260	M	59	0.974
ST0293	M	59	1.189
ST0323	M	60	0.166
ST0341	M	60	0.545
ST0426	M	60	0.193
ST0555	M	53	0.191
ST0517	F	54	1.058
ST0504	F	53	0.155
ST0443	F	55	0.279
ST0483	F	56	1.103
ST0472	F	58	0.165
ST0248	F	59	0.181
ST0260	M	59	1.12
ST0270	F	59	0.628

CHIKV

code	M/F	age	OD
ST0816	F	1	0.148
ST0820	F	1	0.111
ST1166	F	1	0.115
ST1280	F	1	0.143
ST0861	F	3	0.179
ST1236	F	3	0.121
ST1277	F	3	0.071
ST1289	F	3	0.045
ST1145	F	4	0.255
ST1203	F	5	1.222
ST1284	F	6	1.988
ST1186	F	7	0.137
ST1159	F	8	1.932
ST1248	F	8	0.234
ST1122	F	9	0.178
ST1129	F	9	1.781
ST0827	M	1	0.185
ST0899	M	1	0.201
ST1163	M	1	0.124
ST1294	M	1	0.135
ST1295	M	1	0.293
ST0843	M	2	0.174
ST1164	M	2	0.207
ST1197	M	2	0.113
ST1282	M	2	0.061
ST1288	M	2	0.091
ST1296	M	2	0.183
ST0805	M	3	0.179
ST0812	M	4	0.166
ST1244	M	4	0.093
ST0859	M	6	1.186
ST1292	M	6	0.181
ST0878	M	8	1.015
ST1246	M	8	0.128
ST0801	M	4	0.085
ST0850	M	0	0.075
ST1142	F	2	0.095
ST1177	F	3	0.207

code	M/F	age	OD
ST0837	F	10	0.242
ST0858	M	10	2.113
ST0955	M	10	0.159
ST1208	F	10	0.129
ST1245	F	10	0.151
ST1265	F	10	1.828
ST1278	F	10	0.166
ST1119	M	11	0.086
ST1160	F	11	0.114
ST1207	M	11	0.172
ST1215	M	11	1.544
ST1218	F	11	0.078
ST1238	F	11	1.137
ST1240	F	11	0.092
ST1251	F	11	0.082
ST0886	F	12	0.147
ST1216	M	12	0.121
ST1258	M	12	0.156
ST1271	M	12	0.131
ST1299	M	12	0.241
ST1120	M	13	0.084
ST1175	M	13	0.182
ST1210	F	13	1.761
ST1283	F	13	2.147
ST1183	F	14	0.123
ST1213	F	14	0.13
ST1234	M	14	0.167
ST1253	M	14	0.989
ST1255	M	14	1.353
ST1259	M	14	0.108
ST1261	M	14	0.157
ST1262	M	14	0.073
ST1269	F	14	1.746
ST1290	F	15	0.081
ST1266	M	12	1.035
ST0909	F	17	0.13
ST0975	F	17	0.191
ST0992	F	16	0.195

code	M/F	age	OD
ST1404	F	22	0.099
ST1016	F	25	0.09
ST0937	F	26	0.751
ST0944	F	26	0.125
ST1199	F	26	0.145
ST0946	F	27	0.085
ST1468	F	27	0.222
ST1421	F	28	0.102
ST1355	F	29	0.156
ST1484	F	29	0.196
ST0751	M	21	0.13
ST1104	M	25	0.777

code	M/F	age	OD
ST1390	F	32	0.135
ST1040	F	33	0.824
ST1095	F	33	0.147
ST1348	F	33	1.421
ST1370	F	33	1.085
ST0763	F	35	0.183
ST0769	F	35	0.193
ST1061	F	35	0.141
ST1088	F	35	0.111
ST1198	F	35	1.272
ST1362	F	36	0.174
ST1400	F	36	0.137
ST1419	F	36	0.1
ST1467	F	36	0.123
ST1315	F	37	0.092
ST1394	F	37	1.267
ST1406	F	37	0.097
ST0787	F	38	0.148
ST1350	F	38	0.112
ST1398	F	38	1.777
ST1435	F	38	0.101
ST1034	F	39	1.425
ST1347	F	39	0.181
ST1415	F	39	0.907
ST1473	F	39	0.103
ST1077	M	30	0.173
ST1321	M	30	1.02
ST1032	M	34	0.222
ST0988	M	36	1.446
ST1008	M	37	0.123
ST1311	M	37	0.152
ST1336	M	38	0.84
ST1041	M	39	1.191
ST1356	F	35	0.138
ST1372	F	39	0.163
ST1027	F	34	0.243

code	M/F	age	OD
ST1055	F	40	0.188
ST1070	F	40	0.899
ST1352	F	40	0.772
ST1420	F	40	0.156
ST0941	F	41	0.968
ST1457	F	41	0.111
ST1476	F	41	0.154
ST1301	F	43	0.116
ST1395	F	43	0.15
ST1083	F	44	2.284
ST0948	F	45	1.382
ST1059	F	45	1.2
ST1076	F	45	0.386
ST1318	F	45	0.738
ST1343	F	45	0.541
ST1442	F	48	0.59
ST1408	F	49	0.698
ST1056	M	40	0.157
ST1365	M	40	0.14
ST0752	M	41	0.21
ST1049	M	41	1.932
ST1057	M	43	0.096
ST1310	M	43	0.808
ST1021	M	46	0.72
ST1072	M	46	0.093
ST1319	M	46	0.735
ST1333	M	46	0.895
ST1443	M	46	0.113
ST1030	M	47	0.553
ST1323	M	47	0.748
ST1460	M	47	0.353
ST1320	M	48	0.136
ST0800	M	49	0.545
ST1458	M	49	1.314
ST1069	M	46	0.143
ST1424	F	46	0.699
ST1324	M	45	0.57
ST1330	F	40	0.169

code	M/F	age	OD
ST1098	F	50	0.878
ST1375	F	50	0.889
ST1393	F	51	0.078
ST1455	F	51	0.066
ST1465	F	51	0.134
ST0766	F	52	0.157
ST1475	F	53	0.184
ST0788	F	54	0.093
ST1383	F	54	1.206
ST0791	F	55	0.164
ST1003	F	55	0.233
ST1470	F	55	1.011
ST1002	F	56	0.207
ST1367	F	56	0.934
ST1477	F	57	0.736
ST0789	F	58	0.163
ST1464	F	59	0.289
ST1342	M	51	0.149
ST1399	M	51	0.078
ST1036	M	52	0.254
ST1012	M	53	0.145
ST1029	M	53	1.109
ST1091	M	53	0.168
ST1019	M	54	0.911
ST1004	M	54	1.387
ST1099	M	54	0.186
ST1100	M	55	0.166
ST1454	M	55	1.935
ST1052	M	56	1.102
ST1335	M	57	0.13
ST1413	M	57	1.131
ST1001	M	58	1.147
ST1007	M	58	0.624
ST1344	M	58	0.187
ST0762	F	51	0.601
ST0775	M	56	0.292
ST0795	F	58	1.006
ST1466	M	50	0.171

CHIKV

code	M/F	age	OD
LP0057	M	1	0.166
LP0186	M	1	0.146
LP0297	M	3	0.157
LP0020	M	4	0.085
LP0526	M	4	0.159
LP0573	M	4	0.134
LP0622	M	4	0.138
LP0137	M	5	0.167
LP0157	M	5	0.186
LP0212	M	5	0.125
LP0150	M	6	0.108
LP0181	M	6	0.155
LP0205	M	6	0.096
LP0332	M	6	0.181
LP0677	M	7	0.209
LP0702	M	7	0.198
LP0101	M	8	0.174
LP0176	M	8	0.174
LP0151	M	9	0.1
LP0122	F	1	0.154
LP0133	F	1	0.158
LP0210	F	1	0.149
LP0413	F	1	0.175
LP0479	F	1	0.109
LP0048	F	2	0.096
LP0132	F	2	0.21
LP0480	F	2	0.125
LP0138	F	3	0.248
LP0232	F	4	0.276
LP0527	F	4	0.125
LP0740	F	5	0.13
LP0079	F	6	0.144
LP0463	F	6	0.165
LP0665	F	6	0.148
LP0725	F	7	0.104
LP0747	F	7	0.102
LP0162	F	8	0.087
LP0355	F	9	0.098

code	M/F	age	OD
LP0222	F	10	0.107
LP0171	M	12	0.085
LP0165	M	13	0.098
LP0257	M	13	0.089
LP0400	M	13	0.139
LP0149	M	14	0.101
LP0535	M	14	0.073
LP0671	M	15	0.259
LP0703	M	15	0.153
LP0017	M	16	0.138
LP0005	M	17	0.143
LP0378	M	17	0.2
LP0746	M	17	0.171
LP0752	M	17	0.253
LP0487	M	18	0.131
LP0570	M	18	0.084
LP0672	M	19	0.107
LP0511	F	10	0.11
LP0099	F	11	0.073
LP0054	F	12	0.113
LP0627	F	12	0.11
LP0050	F	14	0.084
LP0472	F	14	0.079
LP0510	F	14	0.221
LP0537	F	14	0.116
LP0408	F	16	0.677
LP0586	F	17	0.129
LP0612	F	17	0.202
LP0684	F	17	0.52
LP0574	F	18	0.198
LP0602	F	18	0.098
LP0751	F	18	0.125
LP0522	F	19	0.129
LP0328	F	11	0.135

code	M/F	age	OD
LP0121	M	20	0.072
LP0338	M	20	0.155
LP0730	M	20	0.1
LP0688	M	21	0.075
LP0732	M	21	0.122
LP0019	M	22	0.122
LP0411	M	22	0.102
LP0555	M	22	0.097
LP0695	M	25	0.104
LP0715	M	27	0.166
LP0711	M	28	0.202
LP0728	M	28	0.145
LP0717	M	29	0.219
LP0260	F	20	0.173
LP0379	F	20	0.149
LP0629	F	20	0.186
LP0096	F	22	0.091
LP0219	F	22	0.16
LP0541	F	22	0.149
LP0217	F	24	0.112
LP0638	F	24	2.508
LP0218	F	25	0.107
LP0370	F	25	0.138
LP0427	F	25	0.106
LP0613	F	25	0.117
LP0615	F	27	0.463
LP0513	F	28	0.118
LP0568	F	29	0.205
LP0285	M	23	0.089
LP0407	M	24	0.116
LP0261	M	26	0.142
LP0262	F	26	0.129
LP0607	M	27	0.124
LP0249	M	29	0.13

code	M/F	age	OD
LP0041	M	30	0.129
LP0437	M	30	0.109
LP0547	M	30	0.533
LP0422	M	31	0.122
LP0680	M	31	0.113
LP0674	M	32	0.314
LP0394	M	33	0.161
LP0528	M	33	0.097
LP0736	M	33	0.114
LP0595	M	34	0.567
LP0606	M	34	0.109
LP0714	M	34	0.146
LP0741	M	35	0.229
LP0753	M	35	0.133
LP0387	M	37	0.129
LP0667	M	37	0.099
LP0669	M	37	0.534
LP0294	M	38	0.117
LP0478	M	38	0.244
LP0682	M	38	0.612
LP0689	M	38	0.145
LP0633	F	30	0.499
LP0039	F	31	0.318
LP0388	F	31	0.851
LP0512	F	31	0.133
LP0267	F	32	0.069
LP0225	F	33	0.305
LP0242	F	34	0.128
LP0291	F	34	0.817
LP0072	F	35	1.429
LP0252	F	35	0.193
LP0519	F	35	0.117
LP0538	F	35	0.47
LP0418	F	36	0.095
LP0539	F	37	0.438
LP0066	F	38	0.2
LP0226	F	39	0.181
LP0268	F	39	0.126
LP0430	F	39	0.088

code	M/F	age	OD
LP0001	M	40	0.095
LP0721	M	40	0.204
LP0271	M	41	0.115
LP0030	M	43	1.132
LP0317	M	43	0.2
LP0351	M	43	0.176
LP0420	M	43	0.162
LP0476	M	43	0.101
LP0559	M	43	0.389
LP0655	M	43	0.939
LP0600	M	45	0.138
LP0061	M	46	0.582
LP0286	M	47	0.141
LP0368	M	47	0.708
LP0654	M	47	0.261
LP0565	M	49	0.746
LP0436	F	40	0.143
LP0520	F	40	0.461
LP0660	F	41	0.133
LP0112	F	45	0.061
LP0250	F	45	0.141
LP0455	F	45	1.024
LP0287	F	46	0.12
LP0348	F	46	1.992
LP0111	F	47	0.879
LP0554	F	47	0.546
LP0009	F	48	0.281
LP0107	F	48	0.644
LP0253	F	48	0.508
LP0277	F	49	0.144
LP0357	F	49	0.185
LP0453	F	49	0.189
LP0718	M	48	0.118
LP0093	F	42	1.219

code	M/F	age	OD
LP0319	F	50	1.126
LP0428	M	50	1.13
LP0315	M	52	0.861
LP0007	M	54	0.689
LP0431	M	54	0.285
LP0681	M	54	1.165
LP0450	M	55	0.19
LP0552	M	55	0.24
LP0679	M	55	0.128
LP0469	M	56	0.419
LP0524	M	56	1.282
LP0623	M	56	0.765
LP0405	M	57	1.018
LP0460	M	57	1.22
LP0775	M	57	0.371
LP0763	M	58	0.489
LP0244	M	59	0.221
LP0404	M	59	0.996
LP0777	M	59	0.6
LP0543	F	50	0.82
LP0010	F	51	0.139
LP0228	F	51	1.276
LP0071	F	52	1.936
LP0441	F	52	0.688
LP0675	F	52	0.137
LP0458	F	53	0.275
LP0620	F	53	1.612
LP0278	F	54	0.553
LP0325	F	55	0.462
LP0215	F	56	0.079
LP0762	F	56	0.089
LP0772	F	56	0.177
LP0307	F	58	0.543
LP0769	F	58	1.183

CHIKV

code	M/F	age	OD
AY0118	M	3	0.177
AY0132	M	4	0.292
AY0143	M	4	0.107
AY0079	M	5	0.209
AY0109	M	5	0.157
AY0123	M	5	0.188
AY0155	M	5	0.178
AY0077	M	6	0.286
AY0145	M	6	0.12
AY0160	M	6	0.237
AY0161	M	6	0.322
AY0215	M	7	0.17
AY0187	M	8	0.193
AY0196	M	8	0.12
AY0211	M	8	0.09
AY0251	M	9	0.179
AY0252	M	9	0.208
AY0073	F	3	0.177
AY0097	F	3	0.137
AY0106	F	3	0.166
AY0107	F	4	0.084
AY0111	F	4	0.166
AY0135	F	4	0.147
AY0103	F	5	0.159
AY0119	F	5	0.146
AY0153	F	5	0.094
AY0074	F	6	0.113
AY0142	F	6	0.127
AY0157	F	6	0.092
AY0185	F	7	0.118
AY0205	F	7	0.114
AY0210	F	8	0.114
AY0232	F	9	0.112
AY0240	F	9	0.145

code	M/F	age	OD
AY0235	M	10	0.242
AY0241	M	10	0.081
AY0248	M	10	0.105
AY0253	M	10	0.158
AY0257	M	10	0.165
AY0262	M	10	0.116
AY0263	M	10	0.139
AY0269	M	10	0.135
AY0415	M	12	0.08
AY0416	M	13	0.077
AY0398	M	14	0.207
AY0400	M	14	0.082
AY0394	M	17	0.119
AY0389	M	18	0.174
AY0427	M	18	0.166
AY0610	M	15	0.17
AY0611	M	15	0.238
AY0230	F	10	0.207
AY0233	F	10	0.141
AY0244	F	10	0.14
AY0246	F	10	0.123
AY0249	F	10	0.137
AY0250	F	10	0.146
AY0255	F	10	0.103
AY0259	F	10	0.131
AY0411	F	12	0.111
AY0413	F	12	0.1
AY0417	F	14	0.148
AY0395	F	15	0.108
AY0579	F	16	0.21
AY0373	F	17	0.142
AY0382	F	17	0.126
AY0425	F	17	0.112
AY0580	F	16	0.126
AY0248	M	10	0.144
AY0249	F	10	0.129
AY0740	M	19	0.104
AY0741	M	12	0.109

code	M/F	age	OD
AY0447	M	21	0.116
AY0448	M	21	0.175
AY0446	M	24	0.089
AY0376	M	27	0.167
AY0281	M	20	0.11
AY0296	M	20	0.108
AY0431	M	20	0.143
AY0554	M	21	0.113
AY0573	M	24	0.193
AY0574	M	27	0.309
AY0576	M	27	0.17
AY0279	M	29	0.109
AY0449	M	29	0.123
AY0756	M	21	0.186
AY0629	M	22	0.211
AY0650	M	24	0.232
AY0633	M	27	0.201
AY0387	F	20	0.25
AY0432	F	20	0.079
AY0444	F	21	0.226
AY0392	F	23	0.094
AY0443	F	23	0.09
AY0445	F	23	0.092
AY0577	F	23	0.073
AY0442	F	24	0.129
AY0390	F	28	0.134
AY0380	F	29	0.256
AY0347	F	23	0.102
AY0511	F	23	0.166
AY0287	F	24	0.095
AY0560	F	28	0.971
AY0736	F	21	0.102
AY0758	F	23	0.136
AY0628	F	27	0.229
AY0737	M	29	0.091
AY0744	M	24	0.172

code	M/F	age	OD
AY0458	M	32	0.209
AY0450	M	32	0.286
AY0454	M	33	0.101
AY0529	M	35	0.303
AY0330	M	36	0.139
AY0456	M	36	0.135
AY0495	M	36	0.204
AY0307	M	39	0.09
AY0747	M	30	0.11
AY0645	M	33	0.133
AY0641	M	34	0.127
AY0648	M	34	0.4
AY0724	M	35	0.152
AY0691	M	36	0.175
AY0723	M	36	1.046
AY0742	M	36	0.154
AY0738	M	38	0.24
AY0550	F	30	0.093
AY0393	F	32	0.312
AY0397	F	32	0.206
AY0375	F	33	0.124
AY0391	F	33	0.099
AY0381	F	34	0.135
AY0378	F	35	0.369
AY0396	F	35	0.377
AY0459	F	35	0.158
AY0379	F	36	0.139
AY0372	F	37	0.184
AY0388	F	37	0.148
AY0402	F	36	0.113
AY0359	F	37	0.131
AY0541	F	37	0.155
AY0365	F	39	1.034
AY0642	F	38	0.18
AY0709	F	35	0.032
AY0714	F	31	0.132

code	M/F	age	OD
AY0530	M	43	0.155
AY0318	M	44	1.252
AY0462	M	46	0.209
AY0467	M	46	0.25
AY0501	M	46	0.496
AY0297	M	47	0.125
AY0472	M	47	1.343
AY0315	M	48	0.217
AY0280	M	49	0.096
AY0745	M	40	1.764
AY0746	M	40	0.104
AY0722	M	41	0.175
AY0586	M	42	0.293
AY0698	M	44	0.296
AY0730	M	44	0.492
AY0584	M	47	0.809
AY0732	M	47	0.246
AY0377	F	43	1.283
AY0512	F	40	0.238
AY0271	F	41	0.148
AY0368	F	41	0.269
AY0403	F	41	0.33
AY0545	F	42	0.264
AY0538	F	43	0.225
AY0313	F	44	0.113
AY0340	F	44	0.507
AY0301	F	46	0.371
AY0344	F	47	0.659
AY0321	F	48	1.124
AY0461	F	48	0.115
AY0564	F	48	0.407
AY0473	F	49	0.242
AY0690	F	46	0.647
AY0585	F	49	0.135
AY0557	M	49	0.116
AY0684	F	46	0.339

code	M/F	age	OD
AY0294	M	50	0.129
AY0331	M	50	0.611
AY0470	M	50	0.214
AY0471	M	50	0.478
AY0552	M	50	0.148
AY0549	M	51	0.527
AY0273	M	55	0.774
AY0303	M	57	0.807
AY0591	M	50	0.214
AY0696	M	50	1.407
AY0735	M	50	1.762
AY0654	M	52	0.172
AY0660	M	52	0.691
AY0666	M	54	0.133
AY0672	M	56	1.122
AY0658	M	57	1.413
AY0668	M	57	0.288
AY0385	F	50	0.113
AY0490	F	51	0.181
AY0487	F	52	1.49
AY0489	F	52	1.135
AY0386	F	53	0.09
AY0374	F	59	1.714
AY0486	F	59	0.452
AY0488	F	59	0.325
AY0384	F	51	1.444
AY0326	F	57	0.618
AY0524	F	57	0.991
AY0478	F	58	0.18
AY0675	F	52	0.16
AY0662	F	54	0.114
AY0685	F	55	0.449
AY0665	F	57	0.34
AY0725	F	57	0.261
AY0718	F	52	0.424
AY0728	F	55	0.13

CHIKV

CODE	M/F	AGE	OD
ST0327	M	1	0.114
ST0194	M	2	0.273
ST0019	M	3	0.106
ST0278	F	4	1.328
ST0279	F	6	1.577
ST0280	F	8	1.673
ST0491	M	4	0.056
ST0492	F	5	1.294
ST0439	F	9	0.688
ST0563	F	9	0.073
ST705	M	3	1.725
ST688	M	1	0.069
ST679	M	1	0.198
ST666	F	6	0.994
ST682	M	5	1.44
ST709	M	0	0.059
ST703	M	1	1.195
ST702	M	7	1.698
ST667	F	0	0.065
ST681	F	2	0.082
ST668	F	2	0.052
ST665	M	3	0.145
ST712	M	4	0.235
ST675	F	9	0.083
ST683	F	3	0.06
ST707	F	2	0.382
ST671	F	3	0.915
ST695	F	3	1.143
ST692	M	0	0.104
ST684	F	1	0.051
ST677	F	4	0.115
ST696	M	3	0.181
ST669	F	2	1.322
ST680	F	2	0.186

CODE	M/F	AGE	OD
ST0298	F	10	0.145
ST0411	F	18	0.108
ST0404	F	19	1.284
ST0015	F	19	0.104
ST0024	M	10	0.916
ST0264	M	14	0.154
ST0268	M	14	1.27
ST0289	M	14	0.928
ST0025	M	12	0.061
ST0048	M	19	1.596
ST0193	M	19	0.926
ST0507	M	18	1.117
ST0508	F	18	0.777
ST0509	F	18	1.8
ST0523	F	17	1.167
ST0524	F	17	1.898
ST0525	F	19	1.714
ST0526	F	19	0.148
ST0546	F	19	1.588
ST0553	F	19	1.455
ST0578	M	15	0.07
ST678	F	10	0.082
ST689	F	11	0.133
ST690	F	13	0.201
ST706	M	11	0.721
ST708	M	13	1.563
ST713	M	12	0.239

CODE	M/F	AGE	OD
ST0272	F	20	0.68
ST0029	F	21	1.028
ST0033	F	21	1.807
ST0020	F	24	1.611
ST0208	F	24	1.3
ST0040	F	25	1.554
ST0124	F	25	1.8
ST0140	F	25	0.607
ST0141	F	25	1.001
ST0218	F	25	1.626
ST0206	F	26	1.602
ST0211	F	26	0.172
ST0424	F	26	0.747
ST0430	F	26	0.806
ST0021	F	27	1.187
ST0131	F	28	0.5
ST0207	F	28	0.051
ST0216	F	28	1.708
ST0126	F	29	1.62
ST0135	F	29	1.705
ST0221	M	26	1.521
ST0239	M	26	1.839
ST0306	M	26	0.677
ST0326	M	26	1.559
ST0362	M	26	0.529
ST0285	M	27	1.851
ST0235	M	28	1.809
ST0331	M	28	1.825
ST0367	M	28	0.805
ST0371	M	28	0.864
ST0049	M	21	1.247
ST0348	M	21	0.772
ST0253	M	24	1.492
ST0204	M	25	1.385
ST0309	M	25	0.091
ST0393	M	25	1.349
ST0277	M	26	1.548
ST0284	M	26	0.425

CODE	M/V	AGE	OD
ST0014	F	31	1.248
ST0159	F	31	0.988
ST0246	F	32	0.143
ST0247	F	32	1.692
ST0359	F	33	1.833
ST0360	F	33	1.306
ST0315	F	34	1.39
ST0397	F	34	1.653
ST0421	F	35	0.059
ST0125	F	36	1.868
ST0304	F	37	0.815
ST0313	F	37	1.483
ST0138	F	39	1.725
ST0224	F	39	1.556
ST0433	F	30	1.762
ST0422	F	33	1.808
ST0363	M	30	0.175
ST0399	M	30	0.109
ST0283	M	33	1.005
ST0034	M	34	0.789
ST0243	M	34	1.708
ST0254	M	34	2.023
ST0023	M	35	1.719
ST0400	M	36	1.066
ST0409	M	36	1.82
ST0002	M	37	1.558
ST0257	M	37	1.525
ST0353	M	37	1.627
ST0333	M	38	1.653
ST0249	M	39	0.639
ST0406	M	39	1.635
ST0506	F	33	0.956
ST0510	M	39	1.473
ST0560	M	35	1.948
ST0245	F	31	0.835
ST0290	F	37	1.904

CODE	M/F	AGE	OD
ST0310	F	40	1.312
ST0176	F	41	1.822
ST0220	F	41	1.47
ST0223	F	42	1.321
ST0428	F	42	1.798
ST0312	F	45	1.143
ST0378	F	45	1.777
ST0215	F	46	0.49
ST0320	F	46	0.706
ST0318	F	48	1.154
ST0347	F	48	1.369
ST0425	F	49	1.413
ST0432	F	49	1.301
ST0250	M	40	1.602
ST0382	M	40	1.386
ST0036	M	41	1.424
ST0256	M	41	1.647
ST0405	M	41	1.53
ST0037	M	42	1.454
ST0164	M	45	1.043
ST0394	M	45	2.052
ST0395	M	47	1.73
ST0303	M	49	1.406
ST0580	F	49	1.643
ST0500	M	42	1.787
ST0496	F	45	1.911
ST0437	F	43	1.831
ST0449	F	41	1.889
ST0490	M	40	1.768
ST0522	M	40	0.817
ST0511	M	46	1.823
ST0452	M	43	1.888
ST0434	M	49	1.045
ST0273	F	43	1.33

CODE	M/F	AGE	OD
ST0032	F	51	1.577
ST0162	F	51	1.216
ST0031	F	53	1.759
ST0058	F	53	1.617
ST0232	F	55	1.523
ST0314	F	55	1.724
ST0236	F	56	1.821
ST0060	F	58	1.483
ST0407	F	58	1.7
ST0372	F	59	1.773
ST0292	F	60	1.574
ST0412	F	60	1.511
ST0258	M	50	1.86
ST0375	M	50	1.563
ST0200	M	51	1.684
ST0139	M	54	1.842
ST0255	M	54	1.726
ST0351	M	55	1.371
ST0271	M	56	1.757
ST0061	M	57	1.439
ST0350	M	57	1.625
ST0055	M	59	1.743
ST0064	M	59	1.09
ST0260	M	59	1.897
ST0293	M	59	1.377
ST0323	M	60	1.804
ST0341	M	60	1.38
ST0426	M	60	1.789
ST0555	M	53	2.007
ST0517	F	54	2.017
ST0504	F	53	1.987
ST0443	F	55	1.535
ST0483	F	56	1.242
ST0472	F	58	1.774
ST0248	F	59	1.212
ST0260	M	59	1.945
ST0270	F	59	1.558

DENV

CODE	M/F	AGE	OD
ST0816	F	1	0.148
ST0820	F	1	0.139
ST1166	F	1	0.577
ST1280	F	1	0.723
ST0861	F	3	0.166
ST1236	F	3	0.049
ST1277	F	3	1.307
ST1289	F	3	0.099
ST1145	F	4	2.044
ST1203	F	5	0.173
ST1284	F	6	0.071
ST1186	F	7	0.832
ST1159	F	8	0.111
ST1248	F	8	0.557
ST1122	F	9	1.114
ST1129	F	9	1.708
ST0827	M	1	0.057
ST0899	M	1	0.356
ST1163	M	1	0.152
ST1294	M	1	0.451
ST1295	M	1	0.291
ST0843	M	2	0.139
ST1164	M	2	0.128
ST1197	M	2	0.154
ST1282	M	2	0.042
ST1288	M	2	0.057
ST1296	M	2	1.153
ST0805	M	3	0.256
ST0812	M	4	0.085
ST1244	M	4	0.061
ST0859	M	6	0.059
ST1292	M	6	0.057
ST0878	M	8	0.111
ST1246	M	8	0.123
ST0801	M	4	0.161
ST0850	M	0	0.198
ST1142	F	2	0.148
ST1177	F	3	0.202

CODE	M/F	AGE	OD
ST0837	F	10	0.699
ST0858	M	10	0.083
ST0955	M	10	1.352
ST1208	F	10	0.215
ST1245	F	10	1.485
ST1265	F	10	1.16
ST1278	F	10	0.093
ST1119	M	11	0.233
ST1160	F	11	0.466
ST1207	M	11	1.979
ST1215	M	11	0.114
ST1218	F	11	1.795
ST1238	F	11	1.191
ST1240	F	11	0.141
ST1251	F	11	1.406
ST0886	F	12	0.324
ST1216	M	12	1.276
ST1258	M	12	0.657
ST1271	M	12	0.056
ST1299	M	12	0.066
ST1120	M	13	1.572
ST1175	M	13	0.264
ST1210	F	13	1.322
ST1283	F	13	0.715
ST1183	F	14	2.025
ST1213	F	14	1.65
ST1234	M	14	0.155
ST1253	M	14	0.053
ST1255	M	14	0.215
ST1259	M	14	0.047
ST1261	M	14	0.315
ST1262	M	14	1.11
ST1269	F	14	1.581
ST1290	F	15	1.835
ST1266	M	12	0.114
ST0909	F	17	1.032
ST0975	F	17	1.478
ST0992	F	16	0.077

CODE	M/F	AGE	OD
ST1404	F	22	1.729
ST1016	F	25	0.291
ST0937	F	26	1.938
ST0944	F	26	1.483
ST1199	F	26	1.64
ST0946	F	27	1.225
ST1468	F	27	1.617
ST1421	F	28	1.074
ST1355	F	29	0.893
ST1484	F	29	1.785
ST0751	M	21	1.994
ST1104	M	25	1.199

CODE	M/F	AGE	OD
ST1390	F	32	1.331
ST1040	F	33	1.938
ST1095	F	33	1.711
ST1348	F	33	1.737
ST1370	F	33	0.055
ST0763	F	35	1.966
ST0769	F	35	1.769
ST1061	F	35	1.473
ST1088	F	35	1.567
ST1198	F	35	1.734
ST1362	F	36	0.597
ST1400	F	36	1.343
ST1419	F	36	1.6
ST1467	F	36	1.549
ST1315	F	37	1.416
ST1394	F	37	1.848
ST1406	F	37	1.558
ST0787	F	38	0.954
ST1350	F	38	1.426
ST1398	F	38	1.796
ST1435	F	38	1.655
ST1034	F	39	1.868
ST1347	F	39	1.577
ST1415	F	39	1.551
ST1473	F	39	1.176
ST1077	M	30	1.483
ST1321	M	30	1.543
ST1032	M	34	0.498
ST0988	M	36	1.158
ST1008	M	37	1.884
ST1311	M	37	1.878
ST1336	M	38	1.233
ST1041	M	39	1.716
ST1356	F	35	1.997
ST1372	F	39	1.716
ST1027	F	34	1.777

CODE	M/F	AGE	OD
ST1055	F	40	1.794
ST1070	F	40	1.461
ST1352	F	40	1.126
ST1420	F	40	1.411
ST0941	F	41	1.665
ST1457	F	41	1.718
ST1476	F	41	1.837
ST1301	F	43	1.04
ST1395	F	43	1.25
ST1083	F	44	1.71
ST0948	F	45	1.413
ST1059	F	45	1.392
ST1076	F	45	1.847
ST1318	F	45	1.784
ST1343	F	45	1.192
ST1442	F	48	1.79
ST1408	F	49	0.923
ST1056	M	40	1.363
ST1365	M	40	1.352
ST0752	M	41	1.799
ST1049	M	41	1.89
ST1057	M	43	1.528
ST1310	M	43	1.758
ST1021	M	46	1.66
ST1072	M	46	1.821
ST1319	M	46	1.64
ST1333	M	46	1.66
ST1443	M	46	1.863
ST1030	M	47	1.672
ST1323	M	47	1.327
ST1460	M	47	0.966
ST1320	M	48	1.5
ST0800	M	49	0.936
ST1458	M	49	1.371
ST1069	M	46	0.983
ST1424	F	46	1.913
ST1324	M	45	1.747
ST1330	F	40	1.27

CODE	M/F	AGE	OD
ST1098	F	50	1.945
ST1375	F	50	1.894
ST1393	F	51	1.489
ST1455	F	51	1.722
ST1465	F	51	1.705
ST0766	F	52	1.372
ST1475	F	53	1.382
ST0788	F	54	1.692
ST1383	F	54	1.642
ST0791	F	55	1.383
ST1003	F	55	0.994
ST1470	F	55	1.581
ST1002	F	56	1.747
ST1367	F	56	1.688
ST1477	F	57	0.867
ST0789	F	58	1.884
ST1464	F	59	1.128
ST1342	M	51	1.717
ST1399	M	51	1.667
ST1036	M	52	1.478
ST1012	M	53	1.715
ST1029	M	53	1.991
ST1091	M	53	1.918
ST1019	M	54	1.336
ST1004	M	54	1.172
ST1099	M	54	1.878
ST1100	M	55	1.323
ST1454	M	55	1.708
ST1052	M	56	1.009
ST1335	M	57	1.645
ST1413	M	57	1.753
ST1001	M	58	1.741
ST1007	M	58	1.774
ST1344	M	58	1.709
ST0762	F	51	1.662
ST0775	M	56	1.247
ST0795	F	58	1.666
ST1466	M	50	1.8

DENV

CODE	M/F	AGE	OD
LP0057	M	1	0.31
LP0186	M	1	0.188
LP0297	M	3	1.145
LP0020	M	4	0.166
LP0526	M	4	1.858
LP0573	M	4	0.104
LP0622	M	4	0.101
LP0137	M	5	0.088
LP0157	M	5	0.202
LP0212	M	5	0.084
LP0150	M	6	1.657
LP0181	M	6	1.29
LP0205	M	6	0.08
LP0332	M	6	0.068
LP0677	M	7	0.092
LP0702	M	7	0.115
LP0101	M	8	0.134
LP0176	M	8	0.134
LP0151	M	9	0.126
LP0122	F	1	0.362
LP0133	F	1	0.864
LP0210	F	1	0.156
LP0413	F	1	0.156
LP0479	F	1	0.495
LP0048	F	2	1.369
LP0132	F	2	0.557
LP0480	F	2	0.09
LP0138	F	3	0.438
LP0232	F	4	1.756
LP0527	F	4	0.05
LP0740	F	5	0.189
LP0079	F	6	1.53
LP0463	F	6	0.115
LP0665	F	6	0.08
LP0725	F	7	0.934
LP0747	F	7	1.011
LP0162	F	8	0.067
LP0355	F	9	0.722

CODE	M/F	AGE	OD
LP0222	F	10	0.781
LP0171	M	12	0.083
LP0165	M	13	1.156
LP0257	M	13	0.205
LP0400	M	13	0.856
LP0149	M	14	0.29
LP0535	M	14	1.434
LP0671	M	15	1.464
LP0703	M	15	1.46
LP0017	M	16	1.368
LP0005	M	17	1.385
LP0378	M	17	0.816
LP0746	M	17	1.828
LP0752	M	17	0.369
LP0487	M	18	1.018
LP0570	M	18	0.05
LP0672	M	19	1.551
LP0511	F	10	0.832
LP0099	F	11	0.09
LP0054	F	12	0.74
LP0627	F	12	0.373
LP0050	F	14	0.08
LP0472	F	14	1.746
LP0510	F	14	0.887
LP0537	F	14	0.327
LP0408	F	16	1.308
LP0586	F	17	1.323
LP0612	F	17	1.672
LP0684	F	17	2.168
LP0574	F	18	1.715
LP0602	F	18	0.265
LP0751	F	18	1.731
LP0522	F	19	1.994
LP0328	F	11	0.935

CODE	M/F	AGE	OD
LP0121	M	20	1.78
LP0338	M	20	1.519
LP0730	M	20	0.846
LP0688	M	21	2.026
LP0732	M	21	1.556
LP0019	M	22	1.612
LP0411	M	22	0.911
LP0555	M	22	1.358
LP0695	M	25	1.926
LP0715	M	27	1.198
LP0711	M	28	1.374
LP0728	M	28	1.386
LP0717	M	29	1.783
LP0260	F	20	1.588
LP0379	F	20	0.623
LP0629	F	20	0.266
LP0096	F	22	0.907
LP0219	F	22	1.608
LP0541	F	22	1.665
LP0217	F	24	0.077
LP0638	F	24	2.266
LP0218	F	25	0.94
LP0370	F	25	1.317
LP0427	F	25	0.631
LP0613	F	25	1.839
LP0615	F	27	2.041
LP0513	F	28	2.063
LP0568	F	29	1.669
LP0285	M	23	0.707
LP0407	M	24	2.096
LP0261	M	26	1.757
LP0262	F	26	1.787
LP0607	M	27	1.632
LP0249	M	29	0.905

CODE	M/F	AGE	OD
LP0041	M	30	1.523
LP0437	M	30	1.829
LP0547	M	30	1.432
LP0422	M	31	1.319
LP0680	M	31	1.754
LP0674	M	32	1.907
LP0394	M	33	1.293
LP0528	M	33	1.944
LP0736	M	33	1.318
LP0595	M	34	1.919
LP0606	M	34	1.86
LP0714	M	34	1.755
LP0741	M	35	1.538
LP0753	M	35	1.773
LP0387	M	37	1.627
LP0667	M	37	1.742
LP0669	M	37	1.575
LP0294	M	38	1.147
LP0478	M	38	0.929
LP0682	M	38	1.061
LP0689	M	38	1.821
LP0633	F	30	2.018
LP0039	F	31	1.86
LP0388	F	31	1.285
LP0512	F	31	1.656
LP0267	F	32	1.44
LP0225	F	33	1.591
LP0242	F	34	1.678
LP0291	F	34	0.352
LP0072	F	35	1.599
LP0252	F	35	1.484
LP0519	F	35	2.113
LP0538	F	35	1.99
LP0418	F	36	1.281
LP0539	F	37	1.414
LP0066	F	38	1.834
LP0226	F	39	1.822
LP0268	F	39	1.25
LP0430	F	39	1.36

CODE	M/F	AGE	OD
LP0001	M	40	1.7
LP0721	M	40	1.648
LP0271	M	41	1.758
LP0030	M	43	1.771
LP0317	M	43	1.553
LP0351	M	43	1.373
LP0420	M	43	1.971
LP0476	M	43	1.599
LP0559	M	43	1.374
LP0655	M	43	1.78
LP0600	M	45	1.117
LP0061	M	46	1.685
LP0286	M	47	0.806
LP0368	M	47	1.731
LP0654	M	47	1.073
LP0565	M	49	1.741
LP0436	F	40	1.078
LP0520	F	40	1.349
LP0660	F	41	0.841
LP0112	F	45	1.75
LP0250	F	45	1.717
LP0455	F	45	0.828
LP0287	F	46	1.188
LP0348	F	46	1.78
LP0111	F	47	1.731
LP0554	F	47	2.11
LP0009	F	48	1.645
LP0107	F	48	1.749
LP0253	F	48	1.322
LP0277	F	49	1.657
LP0357	F	49	0.971
LP0453	F	49	0.771
LP0718	M	48	1.701
LP0093	F	42	1.768

CODE	M/F	AGE	OD
LP0319	F	50	1.641
LP0428	M	50	1.785
LP0315	M	52	1.013
LP0007	M	54	1.574
LP0431	M	54	1.323
LP0681	M	54	1.714
LP0450	M	55	1.474
LP0552	M	55	2.033
LP0679	M	55	0.702
LP0469	M	56	1.448
LP0524	M	56	1.849
LP0623	M	56	1.992
LP0405	M	57	1.263
LP0460	M	57	1.683
LP0775	M	57	0.445
LP0763	M	58	1.181
LP0244	M	59	1.812
LP0404	M	59	0.775
LP0777	M	59	0.937
LP0543	F	50	1.97
LP0010	F	51	0.702
LP0228	F	51	1.608
LP0071	F	52	1.259
LP0441	F	52	1.883
LP0675	F	52	1.176
LP0458	F	53	1.32
LP0620	F	53	1.652
LP0278	F	54	1.401
LP0325	F	55	1.804
LP0215	F	56	1.585
LP0762	F	56	1.196
LP0772	F	56	1.491
LP0307	F	58	1.434
LP0769	F	58	1.235

DENV

CODE	M/F	AGE	OD
AY0118	M	3	0.077
AY0132	M	4	0.254
AY0143	M	4	1.515
AY0079	M	5	0.279
AY0109	M	5	0.841
AY0123	M	5	0.407
AY0155	M	5	0.063
AY0077	M	6	0.101
AY0145	M	6	0.982
AY0160	M	6	0.144
AY0161	M	6	0.414
AY0215	M	7	0.875
AY0187	M	8	0.067
AY0196	M	8	0.053
AY0211	M	8	0.056
AY0251	M	9	0.951
AY0252	M	9	0.654
AY0073	F	3	0.114
AY0097	F	3	0.083
AY0106	F	3	0.08
AY0107	F	4	1.376
AY0111	F	4	0.155
AY0135	F	4	0.113
AY0103	F	5	0.169
AY0119	F	5	0.06
AY0153	F	5	0.094
AY0074	F	6	0.65
AY0142	F	6	0.082
AY0157	F	6	0.626
AY0185	F	7	0.102
AY0205	F	7	1.025
AY0210	F	8	0.084
AY0232	F	9	0.209
AY0240	F	9	0.213

CODE	M/F	AGE	OD
AY0235	M	10	0.074
AY0241	M	10	0.071
AY0248	M	10	0.062
AY0253	M	10	1.032
AY0257	M	10	0.851
AY0262	M	10	1.431
AY0263	M	10	0.049
AY0269	M	10	0.056
AY0415	M	12	1.54
AY0416	M	13	0.7
AY0398	M	14	0.389
AY0400	M	14	0.991
AY0394	M	17	0.064
AY0389	M	18	1.062
AY0427	M	18	1.357
AY0610	M	15	0.838
AY0611	M	15	0.09
AY0230	F	10	0.084
AY0233	F	10	0.066
AY0244	F	10	1.11
AY0246	F	10	1.437
AY0249	F	10	0.057
AY0250	F	10	0.048
AY0255	F	10	1.208
AY0259	F	10	0.084
AY0411	F	12	0.849
AY0413	F	12	1.729
AY0417	F	14	1.696
AY0395	F	15	0.109
AY0579	F	16	1.757
AY0373	F	17	0.049
AY0382	F	17	0.884
AY0425	F	17	1.211
AY0580	F	16	1.879
AY0248	M	10	0.062
AY0249	F	10	0.067
AY0740	M	19	1.852
AY0741	M	12	0.977

CODE	M/F	AGE	OD
AY0447	M	21	1.701
AY0448	M	21	0.973
AY0446	M	24	1.069
AY0376	M	27	0.958
AY0281	M	20	1.739
AY0296	M	20	0.076
AY0431	M	20	0.049
AY0554	M	21	1.693
AY0573	M	24	0.177
AY0574	M	27	0.929
AY0576	M	27	1.373
AY0279	M	29	1.425
AY0449	M	29	1.238
AY0756	M	21	1.575
AY0629	M	22	1.846
AY0650	M	24	1.294
AY0633	M	27	1.766
AY0387	F	20	0.112
AY0432	F	20	1.609
AY0444	F	21	1.419
AY0392	F	23	1.94
AY0443	F	23	1.126
AY0445	F	23	1.073
AY0577	F	23	1.671
AY0442	F	24	0.768
AY0390	F	28	0.125
AY0380	F	29	0.473
AY0347	F	23	1.43
AY0511	F	23	1.156
AY0287	F	24	1.745
AY0560	F	28	1.743
AY0736	F	21	0.388
AY0758	F	23	1.985
AY0628	F	27	1.733
AY0737	M	29	1.713
AY0744	M	24	0.168

CODE	M/F	AGE	OD
AY0458	M	32	1.549
AY0450	M	32	1.474
AY0454	M	33	1.47
AY0529	M	35	1.355
AY0330	M	36	1.49
AY0456	M	36	1.546
AY0495	M	36	1.544
AY0307	M	39	1.361
AY0747	M	30	1.203
AY0645	M	33	1.648
AY0641	M	34	1.582
AY0648	M	34	1.628
AY0724	M	35	1.154
AY0691	M	36	1.457
AY0723	M	36	1.805
AY0742	M	36	1.489
AY0738	M	38	1.856
AY0550	F	30	1.329
AY0393	F	32	1.145
AY0397	F	32	1.469
AY0375	F	33	1.343
AY0391	F	33	1.229
AY0381	F	34	1.471
AY0378	F	35	1.558
AY0396	F	35	0.942
AY0459	F	35	1.477
AY0379	F	36	1.433
AY0372	F	37	1.486
AY0388	F	37	1.531
AY0402	F	36	1.243
AY0359	F	37	1.431
AY0541	F	37	1.557
AY0365	F	39	1.47
AY0642	F	38	1.038
AY0709	F	35	1.773
AY0714	F	31	1.899

CODE	M/F	AGE	OD
AY0530	M	43	1.556
AY0318	M	44	0.889
AY0462	M	46	1.747
AY0467	M	46	1.642
AY0501	M	46	0.781
AY0297	M	47	1.019
AY0472	M	47	1.651
AY0315	M	48	1.598
AY0280	M	49	1.401
AY0745	M	40	1.593
AY0746	M	40	1.782
AY0722	M	41	1.422
AY0586	M	42	1.286
AY0698	M	44	1.821
AY0730	M	44	1.538
AY0584	M	47	1.268
AY0732	M	47	1.792
AY0377	F	43	1.747
AY0512	F	40	1.326
AY0271	F	41	1.54
AY0368	F	41	1.109
AY0403	F	41	1.202
AY0545	F	42	1.464
AY0538	F	43	1.246
AY0313	F	44	1.911
AY0340	F	44	1.515
AY0301	F	46	1.466
AY0344	F	47	1.641
AY0321	F	48	1.703
AY0461	F	48	1.733
AY0564	F	48	1.634
AY0473	F	49	1.386
AY0690	F	46	0.793
AY0585	F	49	1.491
AY0557	M	49	1.956
AY0684	F	46	0.799

CODE	M/F	AGE	OD
AY0294	M	50	1.137
AY0331	M	50	1.716
AY0470	M	50	1.292
AY0471	M	50	1.409
AY0552	M	50	1.143
AY0549	M	51	0.328
AY0273	M	55	1.663
AY0303	M	57	1.413
AY0591	M	50	0.801
AY0696	M	50	1.914
AY0735	M	50	1.942
AY0654	M	52	1.521
AY0660	M	52	1.592
AY0666	M	54	1.693
AY0672	M	56	1.475
AY0658	M	57	1.686
AY0668	M	57	1.862
AY0385	F	50	1.53
AY0490	F	51	1.02
AY0487	F	52	1.434
AY0489	F	52	1.749
AY0386	F	53	1.426
AY0374	F	59	1.356
AY0486	F	59	1.608
AY0488	F	59	1.289
AY0384	F	51	1.321
AY0326	F	57	1.407
AY0524	F	57	1.373
AY0478	F	58	1.741
AY0675	F	52	1.17
AY0662	F	54	1.474
AY0685	F	55	1.68
AY0665	F	57	0.723
AY0725	F	57	1.437
AY0718	F	52	1.761
AY0728	F	55	1.3

DENV
